# Detection of Algorithmically Generated Domain Names Using the Recurrent Convolutional Neural Network with Spatial Pyramid Pooling

**DOI:** 10.3390/e22091058

**Published:** 2020-09-22

**Authors:** Zhanghui Liu, Yudong Zhang, Yuzhong Chen, Xinwen Fan, Chen Dong

**Affiliations:** 1 Fujian Key Laboratory of Network Computing and Intelligent Information Processing, College of Mathematics and Computer Science, Fuzhou University, Fuzhou 350116, China; lzh@fzu.edu.cn (Z.L.); 031703135@fzu.edu.cn (Y.Z.); 181700310@fzu.edu.cn (X.F.); dongchen@fzu.edu.cn (C.D.); 2 Key Laboratory of Spatial Data Mining & Information Sharing, Ministry of Education, Fuzhou 350116, China

**Keywords:** domain generation algorithm, algorithmically generated domain name, SMOTE, recurrent convolutional neural network, spatial pyramid pooling

## Abstract

Domain generation algorithms (DGAs) use specific parameters as random seeds to generate a large number of random domain names to prevent malicious domain name detection. This greatly increases the difficulty of detecting and defending against botnets and malware. Traditional models for detecting algorithmically generated domain names generally rely on manually extracting statistical characteristics from the domain names or network traffic and then employing classifiers to distinguish the algorithmically generated domain names. These models always require labor intensive manual feature engineering. In contrast, most state-of-the-art models based on deep neural networks are sensitive to imbalance in the sample distribution and cannot fully exploit the discriminative class features in domain names or network traffic, leading to decreased detection accuracy. To address these issues, we employ the borderline synthetic minority over-sampling algorithm (SMOTE) to improve sample balance. We also propose a recurrent convolutional neural network with spatial pyramid pooling (RCNN-SPP) to extract discriminative and distinctive class features. The recurrent convolutional neural network combines a convolutional neural network (CNN) and a bi-directional long short-term memory network (Bi-LSTM) to extract both the semantic and contextual information from domain names. We then employ the spatial pyramid pooling strategy to refine the contextual representation by capturing multi-scale contextual information from domain names. The experimental results from different domain name datasets demonstrate that our model can achieve 92.36% accuracy, an 89.55% recall rate, a 90.46% F1-score, and 95.39% AUC in identifying DGA and legitimate domain names, and it can achieve 92.45% accuracy rate, a 90.12% recall rate, a 90.86% F1-score, and 96.59% AUC in multi-classification problems. It achieves significant improvement over existing models in terms of accuracy and robustness.

## 1. Introduction

Domain generation algorithms (DGA) provide methods for generating large numbers of pseudo-random domain names using specific parameters such as the date, the time, or text as seeds for random initialization. DGAs are often associated with malicious network behaviors. Recent botnets (e.g., Conficker, Kraken, and Torpig) use DGAs to quickly generate candidate remote command-and-control server domain lists [1,2]. They subsequently redirect normal domain name service (DNS) requests to the botnet [3] for conducting malicious activities, such as distributed denial-of-service attacks, spamming, phishing, and click fraud [4,5,6,7] by establishing communication with the infected host through seemingly valid domain names. Therefore, the effective detection of algorithmically generated domain names is crucial for preventing malicious cyber activities.

In recent years, researchers have proposed several types of models to detect algorithmically generated domain names. Traditional models require manual reverse engineering of the DGA-based malwares, which is time consuming and laborious. The malwares can easily escape detection by changing their DGAs during examination. Therefore, reverse engineering models cannot meet the accuracy and timeliness requirements. Models based on blacklist filtering have a limited coverage of algorithmically generated domain names and cannot adapt to the growth of the malicious domain name set. Models based on traditional statistical machine learning methods have become mainstream in detecting algorithmically generated domain names. These models are based on the analysis of domain names or DNS requests. Models based on the analysis of DNS requests detect algorithmically generated domain names by analyzing the differences in the statistical characteristics of the requested domains, request interval, number of request failures, etc., when sending DNS requests to legitimate domain names and algorithmically generated domain names. Models based on the analysis of domain names detect algorithmically generated domain names by analyzing the differences in the distribution characteristics of characters, words, word lengths, numbers of words, etc., between legitimate and algorithmically generated domain names. The main drawback of these models is that they inevitably require intensive manual feature engineering for building the feature set. When the DGA produces variants, these models require the feature set to be reconstructed. This makes it difficult for the models to adapt to large and frequent changes in the DGAs. Furthermore, models based on the analysis of DNS requests usually rely on third-party credit systems and have very high detection costs.

Neural models have recently achieved remarkable progress in various research fields including computer vision, natural language processing, and network security. Neural models can automatically extract the discriminative category features from domain names and effectively detect algorithmically generated domain names by constructing neural networks with multiple hidden layers. However, neural models rely on large-scale domain name datasets for training and are more susceptible to an imbalanced sample distribution than other models.

To address the aforementioned issues, we propose a model to detect algorithmically generated domain names. Our main contributions are as follows:

- To address the problem of an imbalanced sample distribution, we employ an improved borderline synthetic minority over-sampling algorithm (Borderline-SMOTE) to optimize sample balance in the domain name datasets.
- To address the problem of feature extraction, we propose a hybrid neural network that combines a convolutional neural network, a bi-directional long short-term memory (Bi-LSTM) network, and a spatial pyramid pooling strategy. We first employ a convolutional neural network and Bi-LSTM to extract semantic and contextual features from domain names simultaneously and then refine the contextual representation by utilizing the spatial pyramid pooling strategy to capture multi-scale contextual information from the domain names. Therefore, the features captured by the proposed hybrid neural network have more discriminative power and are less sensitive to noise.
- We conduct extensive experiments and analysis to validate the effectiveness of the sample equalization strategy and the performance of the proposed model RCNN-spatial pyramid pooling (SPP). The experiment results demonstrate that the sample equalization method can provide a benefit to performance, and RCNN-SPP can significantly outperform competing models in terms of accuracy, robustness, and convergence speed.

The remainder of this paper is organized as follows. Section 2 briefly reviews the related works on detecting algorithmically generated domain names. Section 3 provides an overview of the the model and introduces its details. Section 4 presents and discusses the impact of the sample equalization method and the performance of RCNN-SPP and other competing models using several domain name datasets. Finally, Section 5 presents some brief concluding remarks.

## 2. Related Works

Existing models for detecting algorithmically generated domain names are primarily based on reverse engineering, blacklist filtering, statistical machine learning methods, and neural networks.

As an example of a reverse engineering-based model, Plohmann et al. [8] performed a comprehensive measurement study of 43 DGA-based malware families and variants. They also pre-computed all possible domains the DGAs can generate and covered the majority of the known and active DGAs by re-implementing these DGAs. However, reverse engineering of DGA-based malware is resource intensive and time consuming and is incapable of dealing with rapidly evolving DGAs and variants.

Building a blacklist that includes domains and IP addresses involved in malicious operations is a common and simple way of detecting algorithmically generated domain names. Kührer et al. [9] conducted a comprehensive analysis of fifteen public malware blacklists and four blacklists operated by antivirus vendors and found that most blacklists have insufficient coverage of malicious domains and fail to protect against malwares that utilize DGAs. This is because the blacklists can only be updated periodically while the attackers can evade blacklist detection easily by continuously generating different domain names using DGAs.

Other models formulate the detection of algorithmically generated domain names as a classification problem and apply statistical machine learning methods to solve the classification problem. Some models distinguish the algorithmically generated domain names by obtaining discriminative information from DNS requests. Wang et al. [10] proposed a DGA-based botnet detection model called Dbod. Dbod clusters hosts according to the relationship intensity between them and identifies the bot-infected hosts based on the differences in query behavior, such as the query time and count distributions, between compromised and normal hosts. Truong et al. [11] proposed a model to detect domain-flux botnets and DGA-bot infected hosts. The model first locates botnets by analyzing the periodicity characteristics of the DNS requests and then extracts relevant features, such as the length and Shannon entropy of the domain names and the occurrence frequency of n-grams across the domain names, from the stream of DNS requests to distinguish algorithmically generated domain names. Schüppen et al. [12] proposed a novel system to detect DGA-related domain names among arbitrary non-existent domain (NXD) DNS traffic. The system builds a feature set that includes structural, linguistical, and statistical features extracted from the domain names and feeds it into a classifier to identify algorithmically generated domain names. Zang et al. [13] adopted spectral and K-means clustering to cluster the domain names generated by a DGA or its variant and subsequently build a feature set that includes TTL, the distribution of the resolved IP addresses, whois, and historical information from each cluster. Finally, they applied an SVM classifier to identify algorithmically generated domain names. Antonakakis et al. [14] proposed a prototype DGA-bot detection system called Pleiades. Pleiades groups the non-existent domains into clusters according to the groups of hosts that query these domains and then employs an alternating decision tree (ADT) and a hidden Markov model (HMM) to identify algorithmically generated domain names and C&Cservers. These models usually require background information like DNS requests and protocol parsing and rely on a third-party credit system to obtain this information. This is expensive and time consuming in practice.

Considering the remarkable differences between algorithmically generated domain names and human generated domain names in terms of the distribution of alphanumeric characters, domain name length, number of characters, and other features, some models rely on the analysis of domain names to detect algorithmically generated domain names. Yadav et al. [15] analyzed the performance of several statistical metrics including the Kullback–Leibler divergence [16], Jaccard index [17], and Levenshtein edit distance [18] and employed a L1-regularized linear regression model designated as LASSO to identify algorithmically generated domain names. Yang et al. [19] analyzed several types of features including word frequency, parts-of-speech, inter-word correlation, and inter-domain correlations by bi-directional maximum matching and then built an ensemble classifier to identify algorithmically generated domain names. Li et al. [20] proposed a hierarchical model to identify DGA domains. The hierarchical model first classifies the DGA domains from legitimate domains using the decision tree and then groups similar DGAs together to determine the DGA algorithm using the DBSCAN clustering algorithm. Raghuram et al. [21] proposed a generative model by analyzing the probability distribution of characters, words, word lengths, and number of words in human generated domain names. These models require the manual construction of feature sets by users with rich feature-engineering experience. Therefore, they cannot achieve satisfactory results when dealing with new DGAs based on the original feature sets.

Deep neural networks have achieved significant success in various fields including network security. Woodbridge et al. [22] employed a long short-term memory (LSTM) network to learn distinct discriminative features from the character sequences of algorithmically generated and human generated domain names and then applied a binary or multinomial logistic regression classifier to detect DGAs and distinguish one DGA from another. Considering that many DGAs use English wordlists to generate plausibly meaningful domain names, Curtin et al. [23] introduced a novel measure called the smashword score to estimate how closely an algorithmically generated domain name resembles English words and proposed a character-level recurrent neural network to deal with algorithmically generated domain names similar to human generated domain names. Yu et al. [24] proposed a novel criterion for creating a noise-free DGA/non-DGA dataset from real traffic and a CNN-based DGA detection model. However, this model still cannot effectively distinguish between word-based algorithmically generated domain names and legitimate ones. They also studied the problem of how to supply sufficient labeled training data for deep learning-based DGA classifiers [25]. Zeng et al. [26] employed several deep learning models popular in computer vision including Alex, VGG, Squeeze Net, Inception, and ResNet to classify DGA domains and non-DGA domains. These neural models can extract the class features from domain names in an automatic and efficient way. However, they usually rely on large-scale domain name datasets for model training and are sensitive to an imbalanced sample distribution in the training datasets. In addition, considering the diversity and complexity of various DGAs, it is difficult to extract abundant and discriminative class features from domain names using a single type of neural network.

## 3. Proposed Model

### 3.1. Overall Model

**Figure 1:**
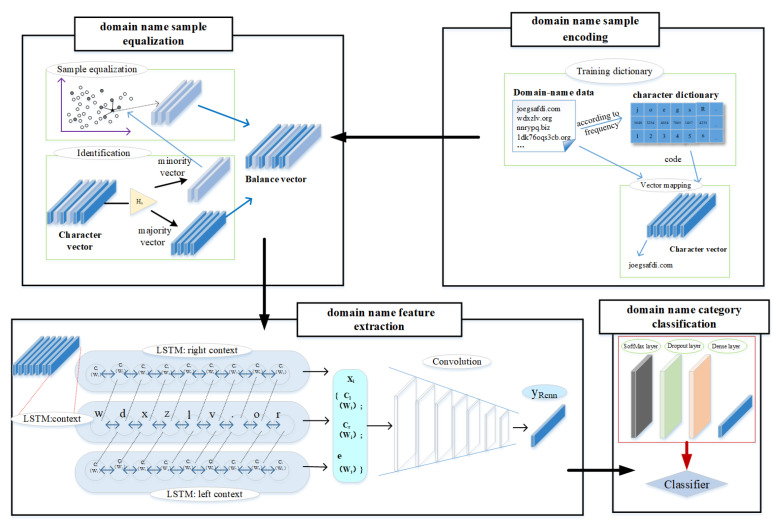
Model framework: The character sequence of the input domain name is processed by the domain name encoding module, the sample equalization module, the domain name representation module, and the classification module.

The model for detecting algorithmically generated domain names is shown in Figure 1 and consists of four modules. The domain name encoding module encodes the character sequence of the input domain name to a sequence of character embedding. To improve the detection accuracy, the sample equalization module then employs the improved Borderline-SMOTE oversampling method to optimize the sample balance between different categories in the dataset. The domain name representation module next employs a hybrid neural network denoted as RCNN-SPP to exploit the semantic information and multi-scale contextual information from domain names and generate the discriminative feature representation for classification. Finally, the feature representation is fed into a softmax layer in the classification module to output the probability distribution over the DGA categories.

### 3.2. Domain Name Encoding

The domain name encoding module encodes the character sequence of an input domain name. First, we create a character dictionary by taking into account the occurrence frequency of each character in the domain name dataset and assign a unique number to each character according to its occurrence frequency. A domain name is then denoted as a character sequence, and its initial vector representation is $v^{\prime }\in R^{l}$ where *l* is the upper bounded length of the domain name and the *i*th element of $v^{\prime }$ is the unique real number assigned to the *i*th character in the character sequence according to the character dictionary. Then, the final fixed-length vector representation $v\in R^{l\times d}$ of a domain name is obtained by mapping each element of $v^{\prime }$ to a vector of $R^{d}$ using a randomly initialized matrix.

### 3.3. Domain Name Sample Equalization

The human generated domain name samples constitute the main part of the dataset. In contrast, the domain names generated by a certain DGA usually constitute only a small proportion of the whole dataset because there is a large number of DGAs. Considering that the neural model requires a large-scale dataset for parameter tuning and is sensitive to sample distribution imbalance [27], the sample equalization module employs the borderline synthetic minority over-sampling algorithm (Borderline-SMOTE) to optimize the sample balance in the dataset.

The SMOTE algorithm is a random oversampling algorithm. The key idea of SMOTE is to select samples from the minority class randomly and synthesize new samples from the minority class by interpolating between nearest samples, thereby increasing the sample size of the minority class and relieving the imbalance in the sample distribution. The Borderline-SMOTE algorithm [28] further addresses the boundary-blur problem of the original SMOTE algorithm by selecting samples located on the class boundary for interpolation instead of selecting them randomly. The details of the domain name sample equalization are described in the following sub-sections.

#### 3.3.1. Identification of Minority Classes

In order to balance the distribution of class samples in the training set of domain name character sequences with class labels, we define the majority class and the minority class. We regard the human generated domain names as belonging to a majority class in the domain name dataset. If the number of samples generated by the malicious domain name algorithm is less than the specified threshold, the corresponding training samples are regarded as a minority class. For the domain names generated by a certain DGA, we use the harmonic mean to determine whether it belongs to a minority or majority class. The harmonic mean equalizes the weights of each class so that it can better reflect the average sample sizes of various DGAs in the dataset.

Suppose that there are *n* different DGAs so that the classes in the dataset can be denoted as $D=\left\{d_{0},d_{1},d_{2},\ldots ,d_{n}\right\}$, where $d_{0}$ refers to the class of human generated domain names and $d_{i}$ refers to the class of the domain names generated by the *i*th DGA. The harmonic mean is defined as follows:

(1) $$H_{n}=\frac{n}{\frac{1}{\left|d_{0}\right|}+\frac{1}{\left|d_{1}\right|}+\frac{1}{\left|d_{2}\right|}+\ldots +\frac{1}{\left|d_{n}\right|}}$$

where $\left|d_{i}\right|{|}_{i=0}^{n}$ is the sample size of class $d_{i}$. If $\left|d_{i}\right|$ is larger than $H_{n}$, $d_{i}$ is regarded as a majority class. Otherwise, $d_{i}$ is regarded as a minority class and requires data enhancement.

#### 3.3.2. Sample Synthesis

After identifying the minority classes in the domain name dataset, the synthesized domain name samples are generated as follows:

Step 1: For each minority class $d_{i}$ identified above and each domain name sample $p_{i,k}{|}_{k=1}^{\left|d_{i}\right|}$ in $c_{i}$, calculate the distance between the vector of $p_{i,k}$ and the other samples in the dataset, and then, build a nearest neighbor set NB(i,k) that consists of the first *K* nearest neighbor samples of $p_{i,k}$.

Step 2: For each domain name sample $p_{i,k}$, if there are less than K/2 samples belonging to $d_{i}$ in NB(i,k), $p_{i,k}$ is regarded as a sample close to the class boundary of $d_{i}$ and is selected as the seed for sample synthesis. Otherwise, if none of the samples in NB(i,k) or more than K/2 samples in NB(i,k) belong to $d_{i}$, $p_{i,k}$ is regarded as a noise sample or a sample far away from the class boundary of $d_{i}$ and will not be selected as a seed for sample synthesis.

Step 3: For each $p_{i,k}$ selected as a seed for sample synthesis, generate a random integer number *s* in the range [1, *K*], and select the first *s* nearest samples from NB(i,k). Then, synthesize *s* domain name samples for class $d_{i}$ by linear interpolation between $p_{i,k}$ and the nearest sample in NB(i,k) via the following formula:

(2) $$synthetic_{j,i,k}=p_{i,k}+r_{j}*diff_{j,i,k}\;j=1,2,\ldots ,s$$

where $synthetic_{j,i,k}$ is a synthesized domain name sample of $d_{i}$, $diff_{j,i,k}$ is the difference between $p_{i,k}$ and the $j_{th}$ nearest sample in NB(i,k), and $r_{j}$ is a random number in the range (0,1) that adjusts the influence of $p_{i,k}$ and the $j_{th}$ nearest sample.

Step 4: Finally, add all the synthesized samples to the original domain name dataset to improve the sample balance.

With the aforementioned analyses, the procedure of sample synthesis for minority classes is summarized in Algorithm 1.
**Algorithm 1** Sample synthesis for minority classes.**Input:** class set $\left\{d_{0},d_{1},d_{2},\ldots ,d_{n}\right\}$, the original domain name dataset *T* **Output:**
*T*1:$H_{n}=n/\left(\frac{1}{a_{1}}+\frac{1}{a_{2}}+\ldots +\frac{1}{a_{n}}\right)$ //calculate the harmonic mean of sample size2:S←{}//initialize the synthesized sample set S as an empty set3:**for**i←1 to n **do**4: **if** $\left|d_{i}\right|<H_{n}$ **then**5:  /*synthesize domain name samples for class $d_{i}$ */6:  **for** K←1 to $\left|d_{i}\right|$ **do**7:   NB(i,k)← choose the first K nearest neighbors of $p_{i,k}$8:   m← calculate the number of samples in NB(i,k) that belong to $d_{i}$9:   **if** 0≤m≤K/2 **then**10:    s← choose a random integer number in the range [1,K]11:    **for** j←1 to *s* **do**12:     $synthetic_{j,i,k}=p_{i,k}+r_{j}*diff_{j,i,k}$
13:     /* add the synthesized domain name sample $synthetic_{j}$ to *S* */14:     $S\leftarrow synthetic_{j,i,k}$
15:    **end for**16:   **end if**17:  **end for**18: **end if**19:**end for**20:T←T∪S // add all the synthesized domain name samples to T

### 3.4. Domain Name Feature Extraction

#### 3.4.1. RCNN-SPP Overview

The most essential task in our model is to learn a distinctive, robust, and discriminative feature representation that can distinguish the differences between human generated and algorithmically generated domain names. Convolutional neural networks (CNNs) and recurrent neural networks (RNNs) are two of the most widely-adopted neural networks for learning discriminative features in many research fields [29]. Both CNNs and RNNs have their own disadvantages. CNNs perform well in capturing the latent semantic information in a domain name. However, they cannot adequately model the semantic correlation and contextual dependency in the character sequence of a domain name, which is critical for learning the differences between human generated and algorithmically generated domain names. In contrast, RNNs perform well in capturing contextual information in the character sequence of a domain name. However, RNNs pay more attention to later characters in the character sequence of a domain name, meaning that later characters have more influence on the feature representation of a domain name than earlier characters, which reduces the effectiveness of RNNs in capturing the semantic information of a domain name.

To address the above issues, we propose a hybrid neural network that combines a convolutional neural network, a bi-directional long short-term memory network, and spatial pyramid pooling (RCNN-SPP). The RCNN-SPP adopts the recurrent convolution neural network (RCNN) proposed by Lai [30] as the backbone neural network. RCNN can effectively learn the semantic and contextual information from domain names while retaining their structure information. Furthermore, we introduce spatial pyramid pooling to acquire multi-scale semantic and contextual information from domain names, which can further improve the feature representations of domain names.

#### 3.4.2. Recurrent Convolutional Neural Network

RCNN-SPP employs a bi-directional recurrent structure to capture forward and backward context for a domain name denoted as $v\in R^{l\times d}$. The forward and backward context of each character is defined as:

(3) $$C_{l}\left(v_{i}\right)=f_{1}\left(W_{l}C_{l}\left(v_{i-1}\right)+W_{sl}e\left(v_{i-1}\right)\right)$$

(4) $$C_{r}\left(v_{i}\right)=f_{1}\left(W_{r}C_{r}\left(v_{i+1}\right)+W_{sr}e\left(v_{i+1}\right)\right)$$

where $C_{l}\left(v_{i}\right)$ and $C_{r}\left(v_{i}\right)$ are the left and right contexts of the character $v_{i}$, respectively, $e(v_{i-1})$ and $e(v_{i+1})$ are the character embedding of the former character $v_{i-1}$ and the latter character $v_{i+1}$, respectively, $W_{l}$, $W_{r}$, $W_{sl}$, and $W_{sr}$ are weight matrices, and $f_{1}$ is a nonlinear activation function. Obviously, the left context of $v_{i}$ is derived from the character embedding and the left context of $v_{i-1}$, while the right context of $v_{i}$ is derived from the character embedding and the right context of $v_{i+1}$ recursively. After obtaining the left and right contexts of each character, the output feature vector of $v_{i}$ is built by cascading the left context of $v_{i}$, the initial character embedding of $v_{i}$, and the right context of $v_{i}$ together, as shown in Equation (5). The output feature vector $x_{i}$ is hence able to represent the contextual information of the entire domain name around $v_{i}$ abundantly.

(5) $$x_{i}=\left[C_{l}\left(v_{i}\right);e\left(v_{i}\right);C_{r}\left(v_{i}\right)\right]$$

After obtaining the latent feature vector of each character in a domain name, the feature representation of a domain name can be represented as $x_{d}=\left[x_{1},x_{2},\ldots ,x_{l}\right]$.

Then, $x_{d}$ is fed into the convolutional layer, and a convolutional filter of size h∗d is employed to perform the convolution operation on $x_{d}$ as shown in the following:

(6) $$o_{i}=F\left(w_{1}.x_{d}\left[i:i+h-1\right]\right)$$

(7) $$c_{i}=f_{2}\left(o_{i}+b_{1}\right)\;i=1,2,\ldots ,s-h+1$$

(8) $$c=[c_{1},c_{2},\ldots c_{s-h+1}]$$

where *F* represents the convolutional filter of size h×d, $w_{1}$ is the weight matrix of the convolutional kernel, $o_{i}$ is the output of the convolution operation, $b_{1}$ is the bias term, $f_{2}$ is the ReLU activation function, and $c_{i}{|}_{i=1}^{s-h+1}$ is the local feature extracted by the convolutional kernel, which constitutes the feature map c of an input domain name.

**Figure 2:**
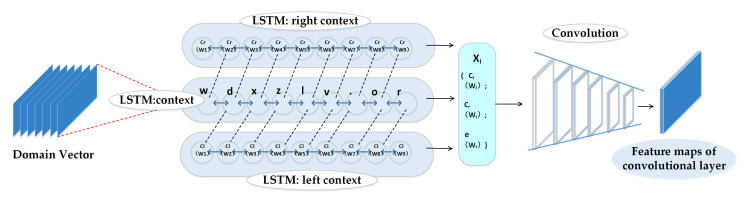
The structure of the recurrent convolutional neural network: The domain vector is first processed by Bi-LSTM and then enters the convolutional layer.

The structure of the recurrent convolutional neural network is shown in Figure 2.

#### 3.4.3. Spatial Pyramid Pooling

**Figure 3:**
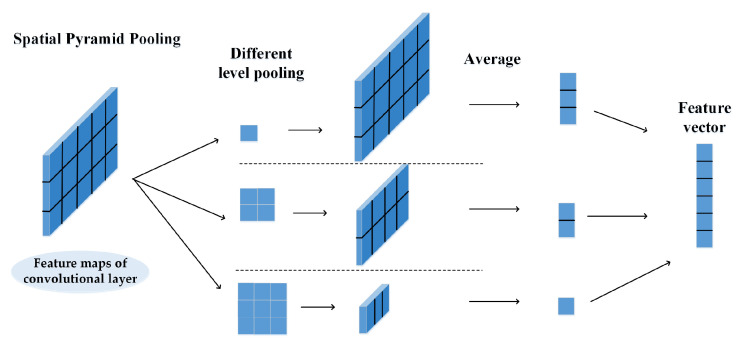
The structure of the spatial pyramid pooling layer: the final feature vector for classification is generated by cascading the feature vectors of different scales of pooling blocks.

A typical CNN always uses a pooling layer to compress the feature map while retaining the discriminative feature information in the feature map. There are generally two types of pooling operations: average pooling and max pooling. However, average pooling and max pooling cannot capture multi-scale feature information from the domain names that is critical for identifying algorithmically generated domain names. Therefore, we employ the spatial pyramid pooling as the pooling layer to capture multi-scale contextual information in the domain names. As shown in Figure 3, pooling is performed on the feature map using n filters with different sizes and strides to obtain feature representations at different scales. These feature vectors obtained by the different scales of pooling blocks are then cascaded to generate the final feature vector for classification.

### 3.5. Output Layer and Parameter Training

The feature vector output by the spatial pyramid pooling layer serves as the final representation. We adopt a softmax layer [31] as the output layer for predicting the DGA class of the input domain name:

(9) $$x=W_{j}o_{j}+b_{j}$$

(10) $$y=softmax\left(x\right)=\frac{exp(x)}{\sum_{j=1}^{\left|D\right|}exp\left(x\right)}$$

where $W_{j}$ and $b_{j}$ are learnable parameters, *D* is the DGA class set, $\left|D\right|$ denotes the number of DGA classes, and $y\in R^{\left|D\right|}$ is the predicted DGA class probability distribution. The DGA class with the highest probability is selected as the DGA class to which the input domain name belongs.

For model training, we employ the cross entropy [32] with the $L_{2}$ regularization term as the loss function:

(11) $$L=\sum_{i=1}^{\left|D\right|}\hat{y_{i}}log\left(y_{i}\right)+\lambda \sum_{\theta \in \Theta }^{}\theta^{2}$$

where $\hat{y_{i}}\in R^{\left|D\right|}$ is a vector that denotes the ground truth, $y_{i}\in R^{\left|D\right|}$ is the predicted DGA class probability distribution, λ is the coefficient of the $L_{2}$ regularization term, and Θ is the collection of all training parameters. We also adopt the dropout strategy to avoid overfitting.

## 4. Experiments

### 4.1. Dataset and Settings

We selected 800,000 DGA domain names from the 360 Netlab OpenData project [33], 750,000 DGA domain names from the Bambenek Consulting feeds [34], and the top 1,000,000 domain names from Alexa [35]. These datasets are widely used for evaluation.

- Dataset from Netlab OpenData: This dataset is provided by 360 Network Security Research Lab and includes 800,000 DGA domain names generated by 50 different types of DGAs collected from botnet and normal Internet traffic data inspected by the mirai scanner. Note that because of the variety of DGAs and very short lifespan of some DGA domain names, some DGAs have only less than 100 domain name samples in this dataset.
- Dataset from Bambenek Consulting: This dataset contains 750,000 DGA domain names generated from time-dependent random seeds by reverse engineering 30 different malware families.
- Dataset from Alexa: Alexa provides a ranking list of popular websites on the Internet. The ranking is calculated by estimating a site’s average number of daily unique visitors and the number of pageviews over the past 3 months. We acquired the top 1 million domain names in the ranking list for 16th May 2018. It is reasonable to assume that these domain names are legitimate domain names (i.e., human generated domain names) because they are related to real sites that have many human visitors and pageviews.

In this study, each experiment was carried out on a large number of domain names randomly selected from the three datasets described above. We selected the DGA domain names (i.e., the negative samples) from Netlab OpenData and Bambenek Consulting, while the legitimate domain names (i.e., the positive samples) were selected from Alexa. In each experiment, we used 80% of the domain name samples for training, 10% for validation, and 10% for testing. It is necessary to note that we adjusted the ratio of legitimate domain names to DGA domain names in accordance with the different purposes of the respective experiments.

In the experiments, we validated our model’s ability to distinguish DGA domain names from legitimate domain names and its ability to determine the DGA classes accurately. Therefore, the detection of DGA domain names is essentially a binary classification or multi-classification task. The ROC curve refers to the receiver operating characteristic curve. AUC is the area under the ROC curve, which is used to measure the performance of algorithms for binary classification problems. For classification tasks, precision, recall, F1-score, and ROC are commonly used as evaluation metrics. Therefore, we used these four metrics to compare the performance of our model with other compared models.

The GPU used in our experiments was an NVIDIA GeForce GTX 1050Ti. The sample equalization method and the RCNN-SPP model were implemented using Keras [36]. The character embedding dimension was 128; the number of feature maps in the convolutional layer was set to 64 and 128. To ensure the accuracy of the experimental results, we ran each experiment 100 times and took the average result.

### 4.2. Results

#### 4.2.1. Performance Analysis of RCNN-SPP

In this section, we conduct several experiments to analyze the binary classification and multi-classification performance of RCNN-SPP. We also study the impact of sample imbalance on the performance of RCNN-SPP and the compared models CNN and LSTM by selecting different domain name samples from the three datasets.

In the first scenario, we studied the performance of RCNN-SPP and the compared models under the condition of a balanced sample class distribution. Therefore, we selected DGA domain name samples from Bambenek Consulting because each DGA class in this dataset has enough samples for model training, whereas some DAG classes in Netlab OpenData have only very few samples.

The total number of domain name samples for the first experiment was 20,000, comprising 20% DGA domain names in 10 DGA classes from Bambenek Consulting with 400 samples for each DGA class and 80% legitimate domain names from Alexa.

**Figure 4:**
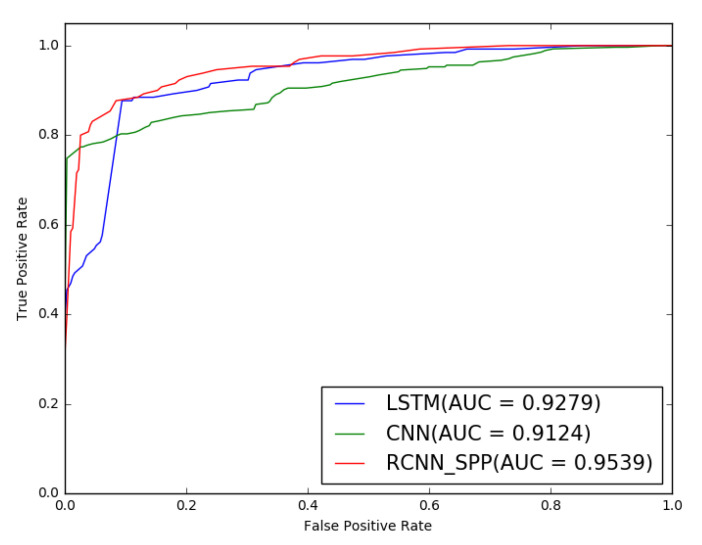
ROC curves of the compared models for binary classification. The area under the ROC curve of RCNN-SPP is the largest, which indicates the best prediction performance.

The first experiment was conducted to validate the performance of RCNN-SPP and the two compared models in binary classification, i.e., in distinguishing DGA domain names from legitimate domain names. The performance result is shown Table 1 and Figure 4. It is obvious that RCNN-SPP achieved superior performance in all evaluation metrics. Compared to LSTM, the precision, recall, F1-score, and AUC of RCNN-SPP are greater by 3.21%, 1.30%, 1.41%, and 2.60%, respectively, representing relative increases of 3.60%, 1.47%, 1.58%, and 2.80%, respectively. The performance improvement can be attributed to the better reflection of the distinctive multi-scale contextual and semantic features between legitimate and DGA domain names in RCNN-SPP due to feeding the output feature representation of the recurrent convolutional neural network to the spatial pyramid pooling layer.

**Figure 5:**
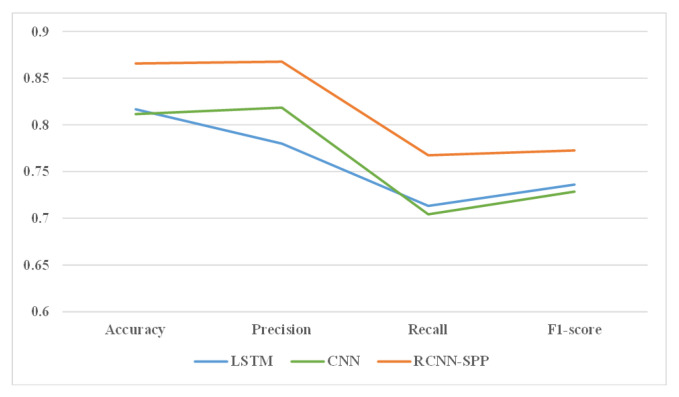
Average performance of the three models in identifying 11 domain classes. RCNN-SPP significantly outperformed the other models in the four evaluation metrics.

**Figure 6:**
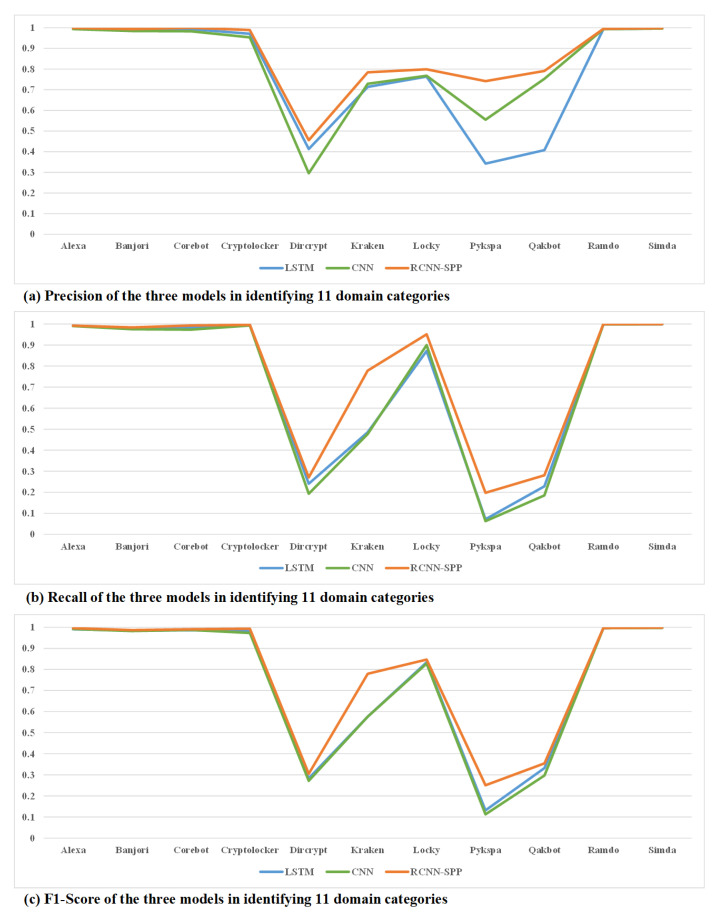
Performance of the three models in identifying 11 domain classes. RCNN-SPP achieved the best performance in all 11 domain classes, especially in the DGA classes of dircrypt and pykspa.

The second experiment was conducted to validate the performance of RCNN-SPP and the two compared models in multi-classification, i.e., accurately determining the DGA class to which a domain name belongs. Figure 5 and Table 2 show the average performance of the three models over 11 domain classes. It can be seen that RCNN-SPP significantly outperformed the other models in all evaluation metrics. Figure 6 and Table 3 shows the detailed performance of the three models in the 11 domain classes. Although the performance gaps between RCNN-SPP and the compared models differ between different classes, RCNN-SPP achieved the best performance in all 11 domain classes, especially in the DGA classes of dircrypt and pykspa. Compared to the LSTM model, the precision, recall, and F1-score of RCNN-SPP in identifying pykspa are greater by 39.96%, 12.58%, and 11.93%, respectively, representing relative increases of 116.68%, 174.00%, and 90.44%, respectively. Compared to the CNN model, the precision, recall, and F1-score of RCNN-SPP in identifying dircrypt are greater by 16.03%, 7.83%, and 3.34%, respectively, representing relative increases of 54.25%, 40.63%, and 12.31%, respectively. It is also worth noting that RCNN-SPP achieved a much greater improvement over the other models in making an accurate decision about the DGA category than it achieved in distinguishing between DGA and legitimate domain names. This is because the combination of a convolutional layer and bi-directional LSTM with a spatial pyramid pooling layer makes RCNN-SPP more capable of extracting distinctive multi-scale contextual dependencies and semantic information in different DGA families.

In the second scenario, we studied the performance of RCNN-SPP and the two compared models under the condition of an imbalanced class sample distribution. Therefore, we selected 10 DGA classes from Bambenek Consulting with 1000 samples for each DGA class and nine DGA classes from Netlab OpenData with 100 samples for each DGA class. We also selected 9100 legitimate domain name samples from Alexa. The total number of domain name samples was 20,000, the same as the first scenario.

**Figure 7:**
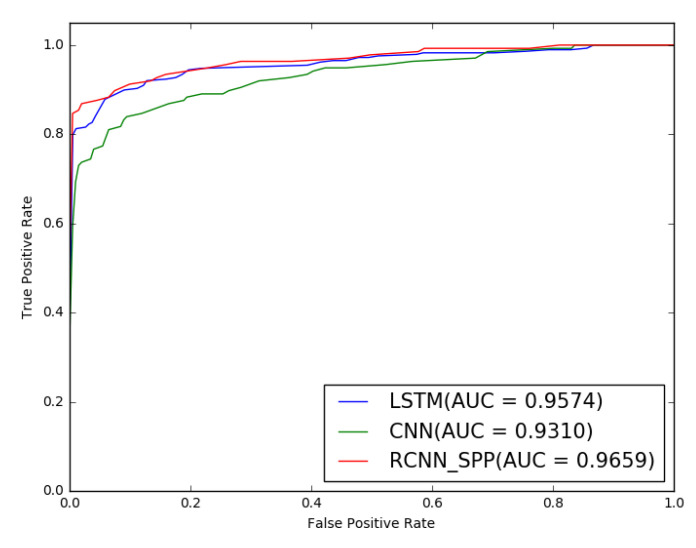
ROC curves of the 20 domain classes for binary classification. The area under the ROC curve of RCNN-SPP is the largest, which indicates the best prediction performance.

Table 4 and Figure 7 show the binary classification performance results of the three models in this scenario. It is obvious that RCNN-SPP achieved superior performance in all the evaluation metrics. Note that all three models achieved better performance than in the first scenario. We attribute the performance improvement to the increase in DGA samples in the training set compared to the first scenario.

**Figure 8:**
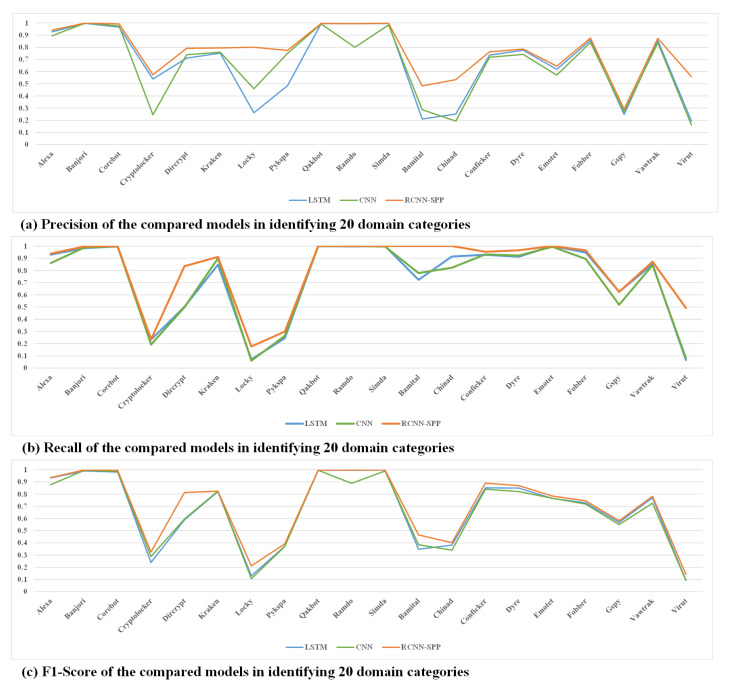
Performance of the compared models in identifying 20 domain classes. RCNN-SPP achieved superior performance in all classes, especially in identifying the DGA classes locky and virut.

Table 5 shows the detailed performance of the three models in 20 domain categories. We can see that RCNN-SPP still achieved superior performance in all classes, especially in identifying the DGA classes locky and virut, as shown in Figure 8. RCNN-SPP also showed more stable performance than the compared models in most DGA classes, which further proves RCNN-SPP’s capability in extracting distinctive class features from different DGA classes. We also found the performance of all three models in the DGA classes from Netlab OpenData to be relatively lower than that on the DGA classes from Bambenek Consulting. This is because the DGA classes from Netlab OpenData suffered from more serious sample imbalance than the DGA classes from Bambenek Consulting.

#### 4.2.2. Analysis of Model Convergence

**Figure 9:**
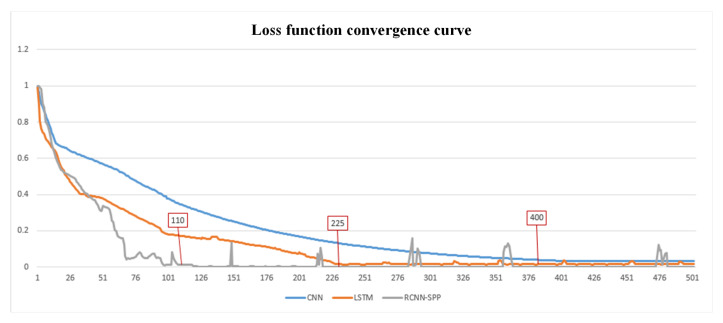
Model performance and loss at each epoch. RCNN-SPP achieves faster convergence speed and smaller loss.

In this section, we discuss the convergence speed of RCNN-SPP and the two compared models based on the second experiment of the first scenario in Section 4.2.1. To illustrate the convergence speed advantage of RCNN-SPP, the classification performance and loss in each epoch for model training are presented in Figure 9. As shown in Figure 9, RCNN-SPP achieved optimal performance faster than CNN and LSTM in the iterative training process. RCNN-SPP achieved the best performance in the 110th epoch, whereas LSTM and CNN achieved their best performance in the 225th and 400th epochs, respectively. We can also observe that the loss of RCNN-SPP became smaller than that of LSTM after the 45th epoch and that RCNN-SPP entered the convergence stage more quickly and steeply. This is because RCNN-SPP uses a recurrent convolutional neural network as the backbone network so that it can extract more distinctive features than CNN and LSTM by combining the advantages of CNN and Bi-LSTM in feature extraction. Furthermore, the spatial pyramid pooling layer also helps RCNN-SPP extract more robust and discriminative multi-scale features. Therefore, the classification performance of RCNN-SPP improves rapidly with a concomitant drop in loss. In summary, RCNN-SPP improves its classification ability rapidly and achieves faster convergence than the compared models.

#### 4.2.3. Analysis of Sample Size

**Figure 10:**
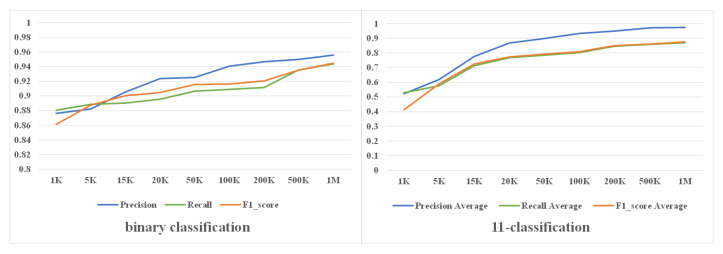
Impact of sample size on model performance. The increase of the sample size can improve the performance of RCNN-SPP and has a greater impact on the performance of multi-classification.

In this section, we discuss the impact of sample size on the performance of RCNN-SPP. We followed the same strategy as the first scenario. The proportions of the samples from the DGA domain names in the 10 DGA classes from Bambenek Consulting and the legitimate domain names from Alexa were fixed at 20% and 80%, respectively, while the total number of domain name samples varied from 1000 to 1,000,000. The binary classification and multi-classification results are shown in Figure 10 and Table 6. From the experiment results, we can see that the sample size has a greater impact on the multi-classification performance than on the binary classification performance. As the sample size increased from 1000 to 1,000,000, the precision, recall, and F1-score of binary classification increased from 88.04%, 86.12%, and 91.75% to 94.35%, 94.45%, and 99.98%, respectively. On the other hand, the average precision, recall, and F1-score of multi-classification increased from 52.24%, 52.86%, and 41.25% to 97.35%, 86.95%, and 87.55%, respectively. This is because the differences between the class feature of DGA domain names and legitimate domain names are significant and RCNN-SPP can learn them from a relatively small sample. In contrast, some DGA classes have similar character distributions, and the differences between their class features are not so obvious. Therefore, RCNN-SPP requires more DGA samples to learn the distinctive features of different DGA classes.

#### 4.2.4. Analysis of Sample Equalization

**Figure 11:**
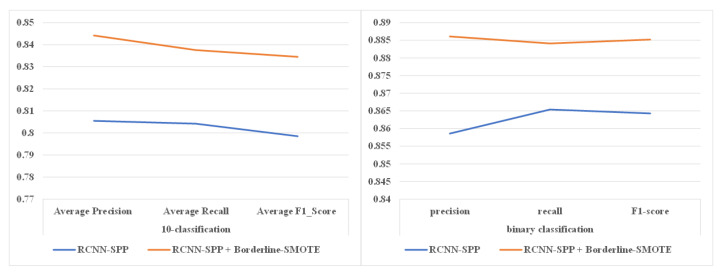
Impact of sample equalization on model performance. The sample equalization can improve the classification performance in both multi-classification and binary classification.

In this subsection, we analyze the impact of sample equalization on the performance of RCNN-SPP under the condition of an imbalanced class sample distribution. We selected nine DGA classes from 360 Netlab OpenData with a total number of 900 domain name samples and 10,000 legitimate domain name samples from Alexa. The classification results are shown in Figure 11 and Table 7. It is obvious that sample equalization can improve the classification performance in both multi-classification and binary classification. Moreover, the performance improvement in multi-classification is greater than that in binary classification. The reason is the same as what we discussed in the previous experiment. RCNN-SPP relies on more DGA samples for learning distinctive features between some DGA categories that have similar patterns.

#### 4.2.5. Analysis of the Spatial Pyramid Pooling

**Figure 12:**
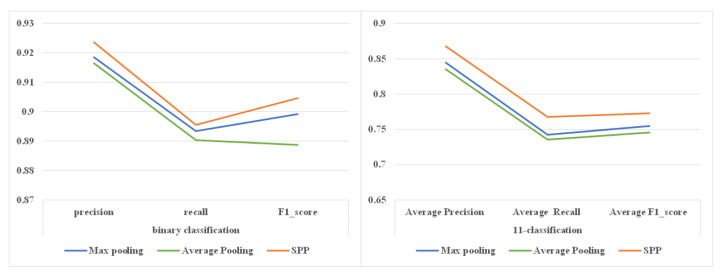
Impact of different pooling strategies on detection performance. Spatial pyramid pooling achieved the best classification performance among the three pooling strategies.

In this section, we discuss the impact of different pooling strategies on the performance of RCNN-SPP based on the dataset used in the first scenario. The experiment results are shown in Figure 12 and Table 8. It is obvious that the average pooling strategy was unable to preserve the category feature details, resulting in the worst classification performance. In comparison with average pooling, max pooling preserved more distinctive feature information implied in the different DGA categories, resulting in a better classification performance than average pooling. Spatial pyramid pooling achieved the best classification performance among the three pooling strategies. Unlike average pooling and max pooling, spatial pyramid pooling can take features at different scales as its input and preserves more discriminative category information in the fusion process. We also found that spatial pyramid pooling achieves more significant performance improvement in multi-classification than in binary classification. The reason is the same as what we discussed in the previous subsection.

## 5. Conclusions

In this paper, we propose a novel model for detecting algorithmically generated domain names. We employ the borderline synthetic minority over-sampling algorithm (SMOTE) to improve sample balance. We also propose a recurrent convolutional neural network to fully exploit the contextual and semantic information in different DGA categories. Furthermore, we adopt the spatial pyramid pooling strategy to refine the category feature representation, which further improves the ability of our model to identify different DGA categories. We also conduct extensive experiments and analysis on several datasets. The experiments demonstrate that our model achieves perfect performance. Future work will consider the optimization of its performance and compare it with the recent work [37,38,39] to evaluate the strength of the model.

## Figures and Tables

**Table 1:** Performance of the three models in identifying domain generation algorithms (DGAs) and legitimate domain names. SPP, spatial pyramid pooling.

Model	Precision	Recall	F1-Score	AUC
LSTM	89.15	88.25	89.05	92.79
CNN	90.24	88.35	87.25	91.24
RCNN-SPP	**92.36**	**89.55**	**90.46**	**95.39**

**Table 2:** Average performance of the three models in identifying 11 domain classes.

Model	Accuracy	Precision	Recall	F1-Score
LSTM	81.66	78.00	71.33	73.62
CNN	81.15	81.84	70.43	72.86
RCNN-SPP	**86.58**	**86.76**	**76.75**	**77.27**

**Table 3:** Performance of the three models in identifying 11 domain classes.

Domain Type	Precision			Recall			F1-Score		
	LSTM	CNN	RCNN-SPP	LSTM	CNN	RCNN-SPP	LSTM	CNN	RCNN-SPP
Alexa	99.55	99.35	**99.85**	99.16	99.05	**99.25**	99.12	99.24	**99.64**
Banjori	99.09	98.42	**99.35**	98.24	97.56	**98.35**	98.52	98.24	**98.73**
Corebot	99.35	98.26	**100**	98.25	97.32	**99.28**	98.65	98.75	**99.08**
Cryptolocker	97.08	95.36	**98.89**	99.42	99.31	**99.65**	98.39	97.31	**99.38**
**Dircrypt**	41.28	29.55	**45.58**	24.11	19.27	**27.10**	28.30	27.14	**30.48**
Kraken	71.36	72.88	**78.41**	48.47	47.68	**77.81**	57.63	57.62	**77.98**
Locky	76.36	76.74	**79.90**	87.20	89.98	**95.11**	83.41	82.79	**84.68**
**Pykspa**	34.21	55.52	**74.17**	7.21	6.25	**19.79**	13.15	11.25	**25.08**
Qakbot	40.75	75.31	**79.05**	22.82	18.51	**28.08**	33.21	29.72	**35.45**
Ramdo	99.32	99.34	**99.48**	99.91	99.92	**99.95**	99.61	99.64	**99.71**
Simda	99.69	99.58	**99.78**	99.91	99.95	**99.98**	99.84	99.77	**99.85**

**Table 4:** Performance in identifying DGA and legitimate domain names across the 20 domain classes.

Model	Precision	Recall	F1-Score	AUC
LSTM	89.32	88.64	89.35	95.74
CNN	90.46	88.67	87.43	93.10
RCNN-SPP	92.45	90.12	90.86	96.59

**Table 5:** Performance of the compared models in identifying 20 domain classes.

Domain Type	Precision			Recall			F1-Score		
	LSTM	CNN	RCNN-SPP	LSTM	CNN	RCNN-SPP	LSTM	CNN	RCNN-SPP
Alexa	92.68	89.43	**94.08**	92.96	86.02	**93.76**	93.21	87.69	**93.51**
Banjori	99.65	**100**	**100**	98.26	98.81	**99.65**	98.95	99.40	**99.83**
Corebot	96.52	97.41	**99.16**	99.66	99.85	**99.92**	98.07	98.61	**99.54**
Cryptolocker	53.96	24.49	**57.11**	23.29	19.25	**23.53**	24.00	28.84	**32.54**
Dircrypt	71.12	73.86	**79.17**	50.26	49.91	**83.52**	58.89	59.57	**81.28**
Kraken	75.19	75.95	**79.40**	84.67	89.91	**91.11**	81.95	82.38	**82.39**
**Locky**	26.15	45.95	**80.00**	7.09	6.03	**17.71**	13.03	10.66	**21.12**
Pykspa	48.48	75.00	**77.42**	24.41	26.44	**29.91**	37.11	36.99	**39.10**
Qakbot	99.45	99.18	**99.55**	**100**	99.88	**100**	99.73	99.53	**99.78**
Ramdo	**99.39**	79.93	99.34	99.63	**100**	**100**	99.51	88.84	**99.67**
Simda	99.54	98.35	**99.69**	99.69	99.56	**99.99**	99.77	98.95	**99.83**
**Bamital**	21.08	28.69	**48.31**	72.33	77.85	**100**	34.82	38.47	**46.46**
**Chinad**	25.13	19.33	**53.40**	91.44	82.28	**100**	38.14	34.05	**40.17**
Conficker	73.49	71.77	**76.19**	92.86	93.14	**95.35**	85.09	84.02	**88.96**
Dyre	77.57	74.14	**78.54**	91.25	92.24	**96.57**	84.95	82.05	**86.91**
Emotet	61.94	57.16	**64.53**	**100**	99.43	**100**	76.49	76.61	**78.44**
Fobber	85.86	83.90	**87.55**	94.74	89.46	**96.51**	72.94	71.83	**74.53**
Gspy	24.75	26.96	**29.15**	**62.57**	51.91	62.43	56.82	54.98	**58.18**
Vawtrak	85.61	83.69	**87.34**	85.04	84.38	**87.24**	77.00	72.59	**78.21**
**Virut**	19.41	16.22	**55.81**	6.52	8.05	**49.16**	9.21	9.16	**14.08**

**Table 6:** Impact of sample size on model performance.

Sample Size	Binary Classification			11 Classification		
	Precision	Recall	F1-Score	Average Precision	Average Recall	Average F1-Score
1 K	87.62	88.04	86.12	52.24	52.86	41.25
5 K	88.23	88.85	88.75	61.89	57.65	58.88
15 K	90.53	89.02	90.02	77.56	71.23	72.45
20 K	92.36	89.55	90.46	86.76	76.75	77.27
50 K	92.52	90.65	91.54	89.82	78.43	79.14
100 K	94.05	90.88	91.62	93.25	80.24	80.86
200 K	94.65	91.15	92.04	94.88	84.55	84.98
500 K	94.97	93.54	93.52	97.08	85.87	86.02
1 M	95.56	94.35	94.45	97.35	86.95	87.55

**Table 7:** Impact of sample equalization on model performance.

Model	Binary Classification			10-Classification		
	Precision	Recall	F1-Score	Average Precision	Average Recall	Average F1-Score
RCNN-SPP	85.86	86.54	86.43	80.55	80.42	79.85
RCNN-SPP + Borderline-SMOTE	88.61	88.41	88.52	84.42	83.76	83.45

**Table 8:** Impact of different pooling strategies on detection performance.

Pooling Strategies	Binary Classification			10-Classification		
	Precision	Recall	F1-Score	Average Precision	Average Recall	Average F1-Score
Average pooling	91.65	89.03	88.87	83.52	73.55	74.56
Max Pooling	91.85	89.34	89.92	84.45	74.23	75.48
SPP	92.36	89.55	90.46	86.76	76.75	77.27
